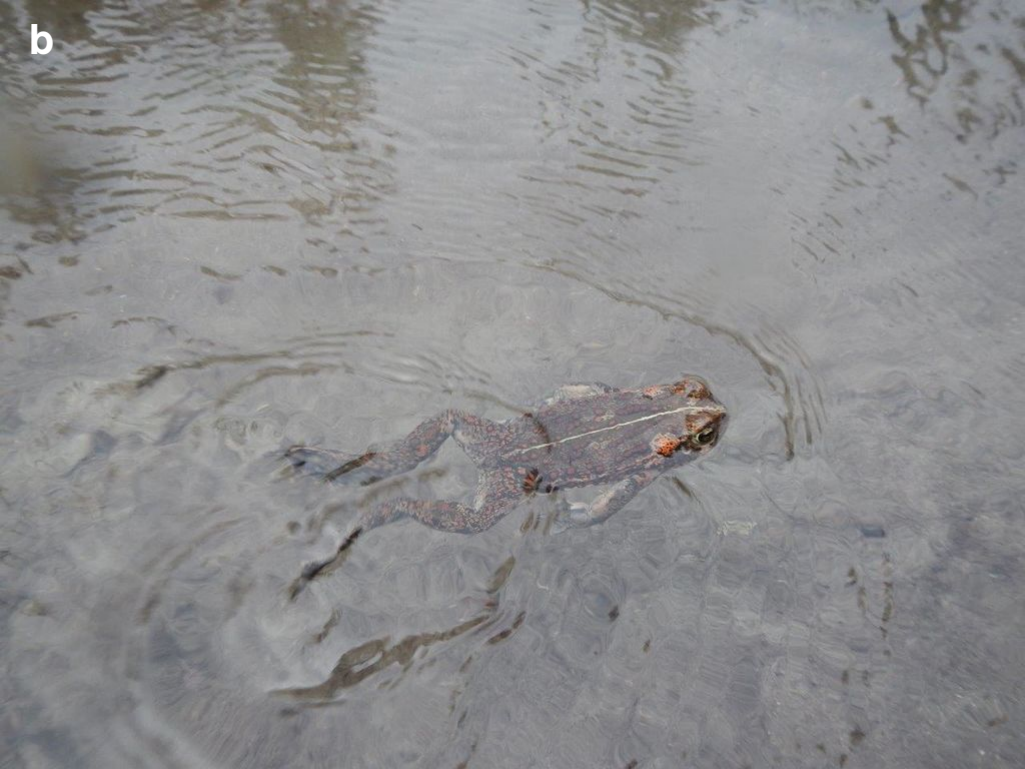# First Canadian record of the water mite *Thermacarus nevadensis* Marshall, 1928 (Arachnida: Acariformes: Hydrachnidiae: Thermacaridae) from hot springs in British Columbia

**DOI:** 10.3897/BDJ.4.e9550

**Published:** 2016-07-22

**Authors:** Jennifer Heron, Cory Sheffield

**Affiliations:** ‡ British Columbia Ministry of Environment, Species Conservation Science Unit, Vancouver, Canada; § Royal Saskatchewan Museum, Regina, Canada

**Keywords:** Hot springs, Acari, Thermacaridae, Canada, British Columbia, DNA barcode

## Abstract

**Background:**

*Thermacarus nevadensis* Marshall, 1928 is an uncommonly collected mite associated with hot spring environments in the western United States. Information on its distribution and ecology are incomplete.

**New information:**

In this paper, we report *Thermacarus nevadensis* from northern British Columbia. These records represent the first of Thermacaridae from Canada, the most northern records of this species in North America, and the most northern records for the family globally. We also provide short notes and images of the habitats in which specimens have been collected in Canada.

## Introduction

Hot spring habitats, as defined by Pentecost et al. (2003), often harbour unique assemblages of species, as the continuous and consistent high-temperature flow and chemical composition of the water provide a stable environment that promotes adaptation to extreme thermal conditions. Hot spring assemblages include thermophiles with specific adaptations and requirements for hot water environments (Brues 1928, Brues 1932, Mitchell 1960, Danks and Williams 1991, Heron 2007) and other species that have developed some tolerance for high temperatures and associated conditions (Brues 1928, Brues 1932, Collins et al. 1976, Darveau et al. 2012) and that can live at the margins of such habitats (Salter 2003).

There are over 115 hot springs in Canada. Most of these are western, with at least 100 reported from British Columbia alone (Salter 2003, Woodsworth and Woodsworth 2014). The invertebrate fauna associated with most hot springs in Canada remains largely uninvestigated (Danks and Williams 1991; though see Salter 2003, Heron 2007 and COSEWIC 2008).

Mites are among the most diverse groups of arthropods, with close to 10 thousand species occurring in Canada (Lindquist et al. 1979), including in springs and other freshwater environments (Danks and Williams 1991). However, despite a high number of water mite species occurring in Canada (Smith 1991, Smith and Cook 1991), none have yet been confirmed from hot spring environments in the country, though Smith et al. (2011) suggested that there could be at least a few species based on fauna found in similar habitats in the adjacent United States.

**Figure 1.**
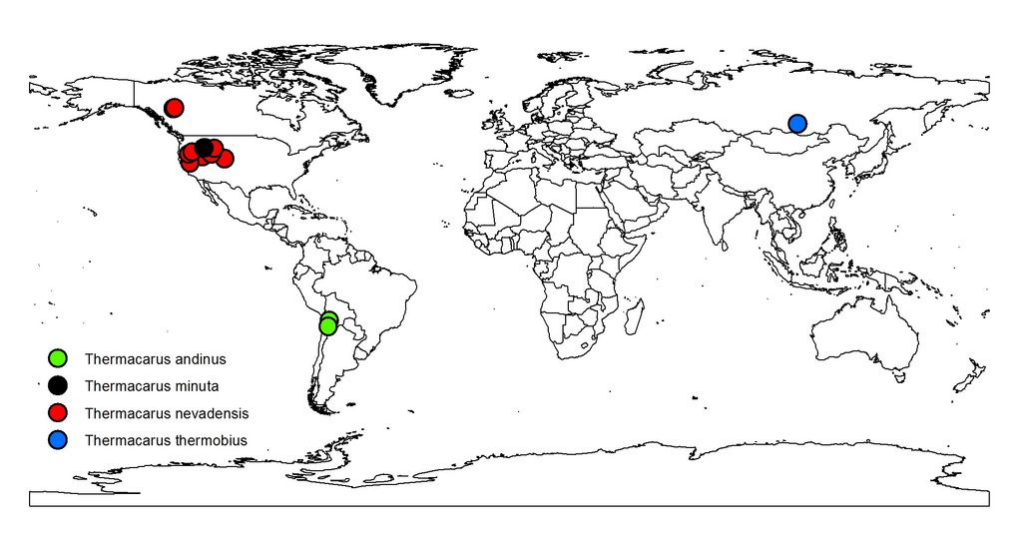
Global distribution of the four known species of *Thermacarus* mites (Thermacadadae).

One family of hot spring-inhabiting mites is Thermacaridae, a monogeneric group with four currently recognized species. The family and genus *Thermacarus* were proposed by Sokolow (1927)​ who discovered the first species, *T. thermobius* Sokolow, 1927 inhabiting 45°C hot spring environments in Lake Baikal (ranging from 51°N - 55°N), Siberia. A year later, *T. nevadensis* Marshall, 1928 was described from a series of specimens collected from two hot springs in Nevada; Valley Hot Springs near Minden, Douglas Co. (ca 39°N) and near Deeth, Elko Co. (ca 41°N) (Marshall 1928), though has subsequently been found in hot springs throughout the northwestern United States (Mitchell 1960, Nyquist 1965, Baker 1985) (Fig. 1). A second, much smaller North American species, *T. minuta* Mitchell, 1963 from hot springs in Loon Creek, Idaho was described by Mitchell (1963). Though *T. nevadensis* was reported from Chile by (Schwoerbel 1987), this material was later described as a fourth species from hot springs in the Southern Hemisphere (Chile, Bolivia), *T. andinus* Martin and Schwoerbel, 2002 (Martin and Schwoerbel 2002).

In this paper, we report the first records of Thermacaridae from Canada. These Canadian records also represent the most northern occurrences for this family known globally.

## Materials and methods

### Survey Sites in Northern British Columbia

**Figure 2.**
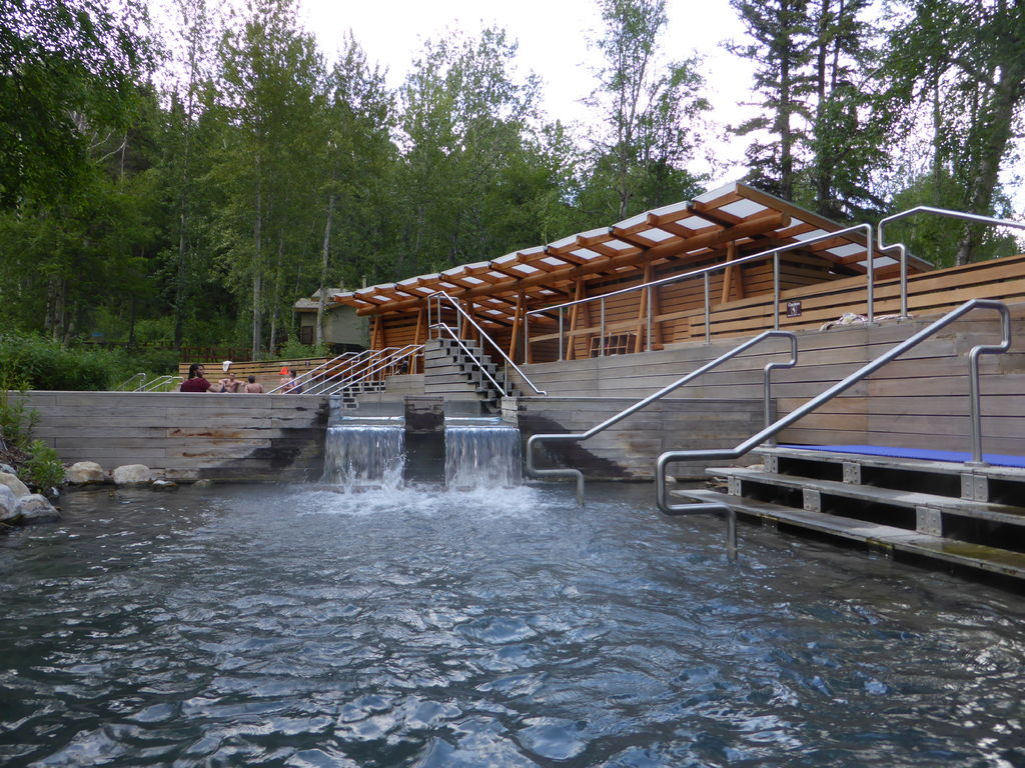
The public hot spring at Alpha Pool in Liard River Hot Springs Provincial Park in northeastern British Columbia. Mites were collected on the undeveloped border of the pool to the left of the photo. Photo by C. Sheffield.

**Figure 3.**
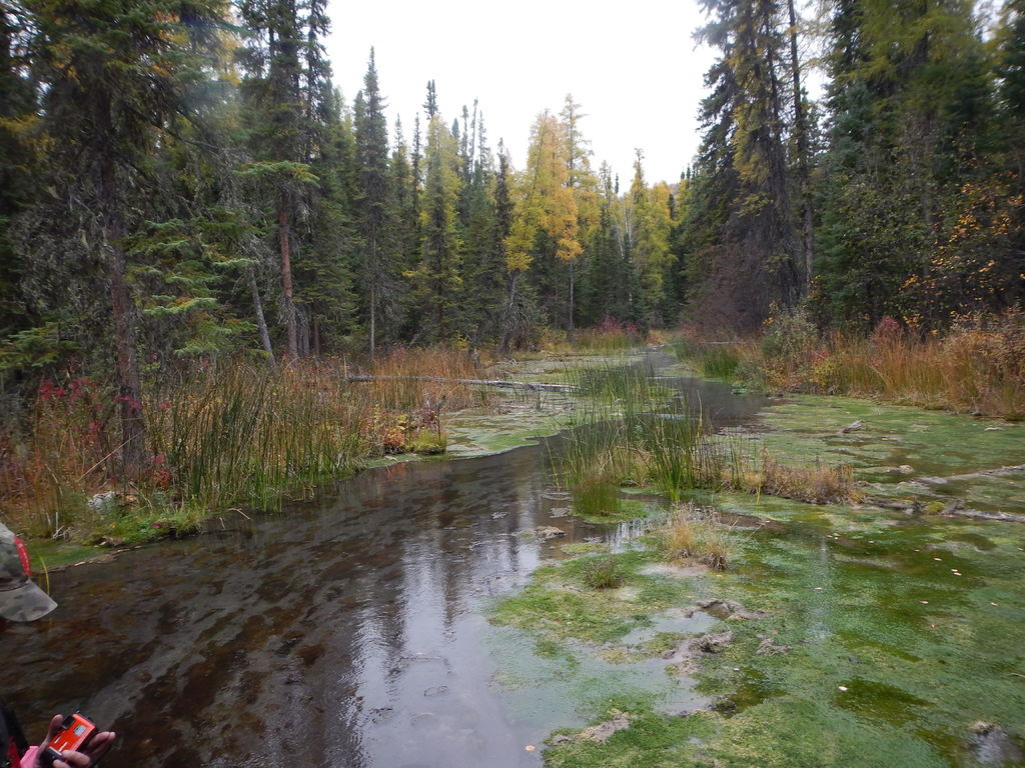
Alpha Stream at Liard River Hot Springs Provincial Park in northeastern British Columbia. Photo by J. Heron.

The Liard River hot springs and the extensive hot spring swamps are located at kilometre 765 of the Alaska Highway in northeastern British Columbia within Liard River Hot Springs Provincial Park (59.431, -126.1), and are the only known location for Hotwater Physa (*Physella wrighti* Te and Clarke, 1985), an endangered freshwater pulmunate snail (Heron 2007, COSEWIC 2008). Within the Liard hot springs complex, the main hot spring feeds Alpha Pool, a developed, publicly accessible pool with year-round access and recreational use (Fig. 2) that flows into the Alpha Stream which travels a few hundred metres before emptying into a large swamp complex (Fig. 3). The water temperature in Alpha Pool ranged from 42°C to 52°C; Alpha Stream temperatures are cooler, ranging from 32°C to 35°C degrees.

**Figure 4.**
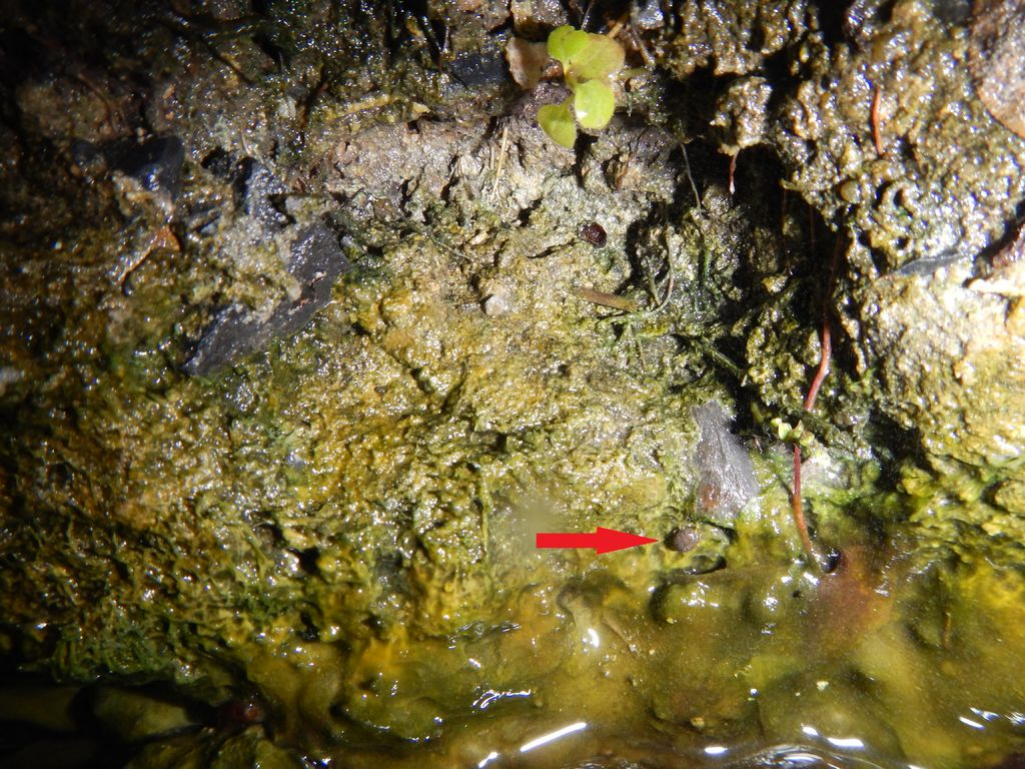
Hot spring mite, *Thermacarus nevadensis* (red arrow) on an algal mat in Alpha Pool at Liard River hot springs in Liard River Hot Springs Provincial Park. Photo by J. Heron.

**Figure 5.**
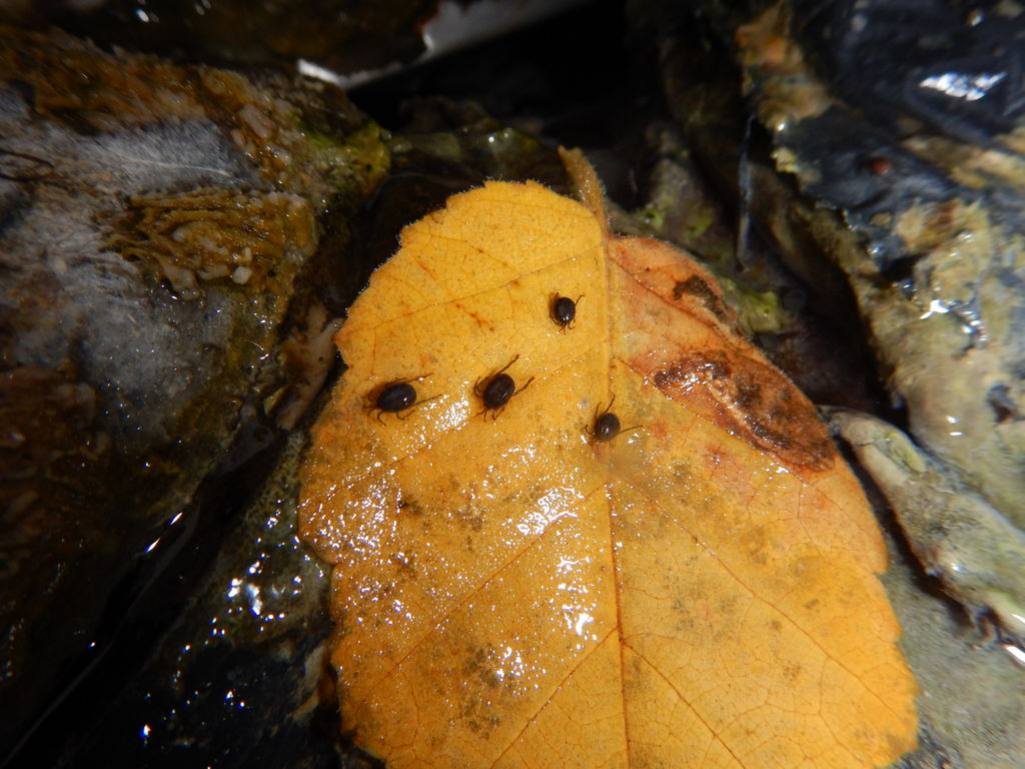
Hot spring mites, *Thermacarus nevadensis* from Alpha Stream at Liard River hot springs in Liard River Hot Springs Provincial Park. Photo by J. Heron.

The natural margins of Alpha Pool and Alpha Stream had extensive algal growth, both just above the surface and under the water, and many mites were observed crawling on these mats within 10 cm above the water-air interface in 2014 (Fig. 4; Mitchell (1960) reported that mites will burrow into these mats, though this was not observed during this study). At Alpha Pool, mites could be individually observed and collected from the algal mats. In 2008, 2015 and 2016 mites were also collected from Alpha Stream (Fig. 5). Subsequent surveys in other areas of Liard River Hotsprings Provincial Park in September 2015 and March 2016 documented the mite within the Delta/Epsilon and Gamma springs and thermal swamps of the park.

Several other hot springs in northeastern British Columbia were also examined during surveys conducted by the British Columbia Ministry of Environment to look for Hotwater Physa (Heron 2007, COSEWIC 2008) and other hot spring fauna. An undeveloped hot spring within the Grayling River Hot Springs Ecological Reserve (59.61612, -125.54283) was visited in 2014. This hot spring is in a remote, protected area in northeastern British Columbia (Fig. 6), with temperatures ranging from 38.9°C (pool margins) to 43.5°C (near one of the sources); there are additional springs in the area that are likely hotter. Mites were not directly sought or observed within this hot spring complex, but specimens were collected during routine sampling using aqualtic nets within the algal mats floating on the water surface.

**Figure 7.**
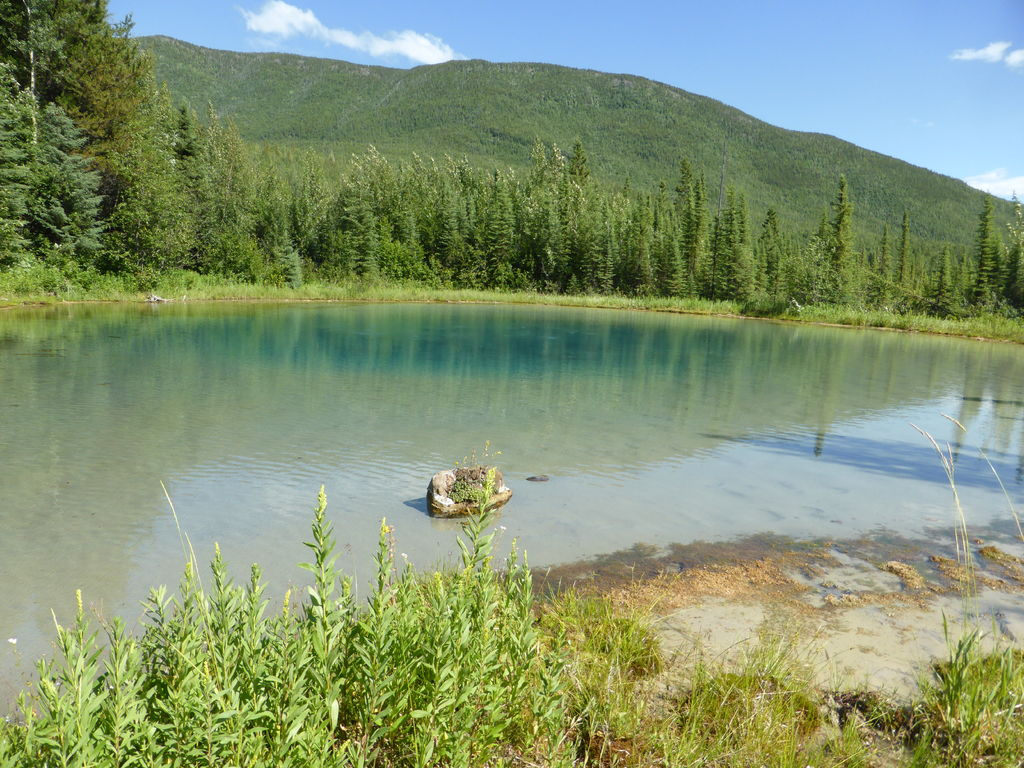
The main pool at Deer River hot spring within Liard River Hot Springs Provincial Park. This site had cooler water and lacked the dense algal mats. No mites were collected in this location. Photo by C. Sheffield.

The Deer River hot springs (59.504163, -125.956703) were also visited in 2014 and 2016, and are also located within Liard River Hot Springs Provincial Park (Fig. 7). The main pool was significantly cooler (32°C) than the other sites, and was without the dense algal mats. No mites were collected at this site with nets in either visit, though surrounding pools were not surveyed.

### DNA Barcoding

To contribute DNA barcodes to the ongoing Barcodes of Life campaign, tissue samples were taken from arthropods from all hot springs surveyed, including mites from both the Liard River and Grayling sites, and then sent to be processed and sequenced for the DNA barcode region of cytochrome c oxidase subunit 1 (Hebert et al. 2003) at the Biodiversity Institute of Ontario, Guelph, Ontario. DNA sequences, specimen photographs (Fig. 8), and all associated data are now in the Barcodes of Life Data (BOLD) System, Project THRCA (Canadian *Thermacarus* Mites), with the following BankIt and GenBank accession numbers: BankIt1918658 RBCMI1034-14.COI-5P KX232857, BankIt1918658 RBCMI1033-14.COI-5P KX232858, BankIt1918658 RBCMI1032-14.COI-5P KX232859, BankIt1918658 RBCMI1031-14.COI-5P KX232860, BankIt1918658 RBCMI1030-14.COI-5P KX232861, BankIt1918658 RBCMI1029-14.COI-5P KX232862. The specimens have been assigned Barcode Index Number (BIN) BOLD:ACR1240 (Ratnasingham and Hebert 2013).

All specimens examined in this study are deposited in the Royal Saskatchewan Museum (RSKM) entomology collection (Regina, SK). Upon completion, voucher material will also be deposited in the Royal British Columbia Museum (RBCM, Victoria, BC), the E.H. Strickland Entomological Museum, University of Alberta (Edmonton, AB), and the Canadian National Collection of Insects, Arachnids and Nematodes (CNC, Ottawa, ON).

## Taxon treatments

### Thermacarus nevadensis

Marshall, 1928

http://www.catalogueoflife.org/col/details/species/id/6f12cc34c519b77642a7949412615f3a/source/tree

#### Materials

- **Type status:** Other material. **Occurrence:** catalogNumber: RSKM_ENT_E-119203; recordedBy: J. Heron; individualCount: 1; sex: female; lifeStage: adult; **Taxon:** scientificName: Thermacarus nevadensis; kingdom: Animalia; phylum: Arthropoda; class: Arachnida; order: Trombidiformes; family: Thermacaradiae; genus: Thermacarus; specificEpithet: nevadensis; scientificNameAuthorship: Marshall; **Location:** country: Canada; stateProvince: British Columbia; locality: Liard River Hot Springs Provincial Park; decimalLatitude: 59.427028; decimalLongitude: -126.091976; georeferenceProtocol: label; **Identification:** identifiedBy: C.S. Sheffield; dateIdentified: 2014; **Event:** eventDate: 09/25/2008; **Record Level:** language: en; institutionCode: RSKM; collectionCode: ENT; basisOfRecord: PreservedSpecimen
- **Type status:** Other material. **Occurrence:** occurrenceDetails: http://www.boldsystems.org/index.php/API_Public/specimen?bin=BOLD:ACR1240; recordNumber: CCDB-22802 D08; recordedBy: C. Sheffield, J. Heron; individualID: CCDB-22802 D08; individualCount: 1; associatedMedia: http://www.boldsystems.org/pics/JHTHE/CCDB-22802_D08+1413482324.jpg; **Taxon:** phylum: Arthropoda; class: Arachnida; order: Trombidiformes; **Location:** country: Canada; stateProvince: British Columbia; decimalLatitude: 59.431; decimalLongitude: -126.1; **Identification:** identifiedBy: Cory S. Sheffield; **Event:** eventDate: 2014-07-08
- **Type status:** Other material. **Occurrence:** occurrenceDetails: http://www.boldsystems.org/index.php/API_Public/specimen?bin=BOLD:ACR1240; recordNumber: CCDB-22802 D09; recordedBy: C. Sheffield, J. Heron; individualID: CCDB-22802 D09; individualCount: 1; associatedMedia: http://www.boldsystems.org/pics/JHTHE/CCDB-22802_D09+1413482324.jpg; **Taxon:** phylum: Arthropoda; class: Arachnida; order: Trombidiformes; **Location:** country: Canada; stateProvince: British Columbia; decimalLatitude: 59.431; decimalLongitude: -126.1; **Identification:** identifiedBy: Cory S. Sheffield; **Event:** eventDate: 2014-07-08
- **Type status:** Other material. **Occurrence:** occurrenceDetails: http://www.boldsystems.org/index.php/API_Public/specimen?bin=BOLD:ACR1240; recordNumber: CCDB-22802 D10; recordedBy: C. Sheffield, J. Heron; individualID: CCDB-22802 D10; individualCount: 1; associatedMedia: http://www.boldsystems.org/pics/JHTHE/CCDB-22802_D10+1413482324.jpg; **Taxon:** phylum: Arthropoda; class: Arachnida; order: Trombidiformes; **Location:** country: Canada; stateProvince: British Columbia; decimalLatitude: 59.431; decimalLongitude: -126.1; **Identification:** identifiedBy: Cory S. Sheffield; **Event:** eventDate: 2014-07-08
- **Type status:** Other material. **Occurrence:** occurrenceDetails: http://www.boldsystems.org/index.php/API_Public/specimen?bin=BOLD:ACR1240; recordNumber: CCDB-22802 D11; recordedBy: C. Sheffield, J. Heron; individualID: CCDB-22802 D11; individualCount: 1; associatedMedia: http://www.boldsystems.org/pics/JHTHE/CCDB-22802_D11+1413482324.jpg; **Taxon:** phylum: Arthropoda; class: Arachnida; order: Trombidiformes; **Location:** country: Canada; stateProvince: British Columbia; decimalLatitude: 59.431; decimalLongitude: -126.1; **Identification:** identifiedBy: Cory S. Sheffield; **Event:** eventDate: 2014-07-08
- **Type status:** Other material. **Occurrence:** occurrenceDetails: http://www.boldsystems.org/index.php/API_Public/specimen?bin=BOLD:ACR1240; recordNumber: CCDB-22802 D12; recordedBy: C. Sheffield, J. Heron; individualID: CCDB-22802 D12; individualCount: 1; associatedMedia: http://www.boldsystems.org/pics/JHTHE/CCDB-22802_D12+1413482324.jpg; **Taxon:** phylum: Arthropoda; class: Arachnida; order: Trombidiformes; **Location:** country: Canada; stateProvince: British Columbia; decimalLatitude: 59.616; decimalLongitude: -125.543; **Identification:** identifiedBy: Cory S. Sheffield; **Event:** eventDate: 2014-07-08
- **Type status:** Other material. **Occurrence:** occurrenceDetails: http://www.boldsystems.org/index.php/API_Public/specimen?bin=BOLD:ACR1240; recordNumber: CCDB-22802 E01; recordedBy: C. Sheffield, J. Heron; individualID: CCDB-22802 E01; individualCount: 1; associatedMedia: http://www.boldsystems.org/pics/JHTHE/CCDB-22802_E01+1413483300.jpg; **Taxon:** phylum: Arthropoda; class: Arachnida; order: Trombidiformes; **Location:** country: Canada; stateProvince: British Columbia; decimalLatitude: 59.616; decimalLongitude: -125.543; **Identification:** identifiedBy: Cory S. Sheffield; **Event:** eventDate: 2014-07-08
- **Type status:** Other material. **Occurrence:** recordedBy: J. Heron; individualCount: 1; lifeStage: adult; preparations: in ethanol; **Taxon:** scientificName: Thermacarus nevadensis Marshall, 1928; kingdom: Animalia; phylum: Arthropoda; class: Arachnida; order: Trombidiformes; family: Thermacaradiae; genus: Thermacarus; specificEpithet: nevadensis; **Location:** continent: North America; country: Canada; stateProvince: British Columbia; locality: Liard River Hot Springs Provincial Park, Alpha Stream; decimalLatitude: 59.42955; decimalLongitude: -126.10016; **Identification:** dateIdentified: 2016; **Event:** eventDate: 03/23/2016; **Record Level:** language: en; institutionCode: RSKM; collectionCode: ENT; basisOfRecord: PreservedSpecimen
- **Type status:** Other material. **Occurrence:** recordedBy: J. Heron; individualCount: 1; lifeStage: adult; preparations: in ethanol; **Taxon:** scientificName: Thermacarus nevadensis Marshall, 1928; kingdom: Animalia; phylum: Arthropoda; class: Arachnida; order: Trombidiformes; family: Thermacaradiae; genus: Thermacarus; specificEpithet: nevadensis; **Location:** continent: North America; country: Canada; stateProvince: British Columbia; locality: Liard River Hot Springs Provincial Park, Alpha Stream; decimalLatitude: 59.42955; decimalLongitude: -126.10016; **Identification:** dateIdentified: 2016; **Event:** eventDate: 03/23/2016; **Record Level:** language: en; institutionCode: RSKM; collectionCode: ENT; basisOfRecord: PreservedSpecimen
- **Type status:** Other material. **Occurrence:** recordedBy: J. Heron; individualCount: 1; lifeStage: adult; preparations: in ethanol; **Taxon:** scientificName: Thermacarus nevadensis Marshall, 1928; kingdom: Animalia; phylum: Arthropoda; class: Arachnida; order: Trombidiformes; family: Thermacaradiae; genus: Thermacarus; specificEpithet: nevadensis; **Location:** continent: North America; country: Canada; stateProvince: British Columbia; locality: Liard River Hot Springs Provincial Park, Alpha Stream; decimalLatitude: 59.42955; decimalLongitude: -126.10016; **Identification:** dateIdentified: 2016; **Event:** eventDate: 03/23/2016; **Record Level:** language: en; institutionCode: RSKM; collectionCode: ENT; basisOfRecord: PreservedSpecimen
- **Type status:** Other material. **Occurrence:** recordedBy: J. Heron; individualCount: 1; lifeStage: adult; preparations: in ethanol; **Taxon:** scientificName: Thermacarus nevadensis Marshall, 1928; kingdom: Animalia; phylum: Arthropoda; class: Arachnida; order: Trombidiformes; family: Thermacaradiae; genus: Thermacarus; specificEpithet: nevadensis; **Location:** continent: North America; country: Canada; stateProvince: British Columbia; locality: Liard River Hot Springs Provincial Park, Alpha Pool; decimalLatitude: 59.427028; decimalLongitude: -126.091976; **Identification:** dateIdentified: 2016; **Event:** eventDate: 09/25/2008; **Record Level:** language: en; institutionCode: RSKM; collectionCode: ENT; basisOfRecord: PreservedSpecimen

#### Distribution

Canada, United States

#### Notes

Thermacaridae can be recognized using the keys of Cook (1974) and Walter et al. (2009). Additional detailed descriptions and images of *Thermacarus nevadensis* can be found in Marshall (1928) and Baker (1985).

## Discussion

**Figure 9a.**
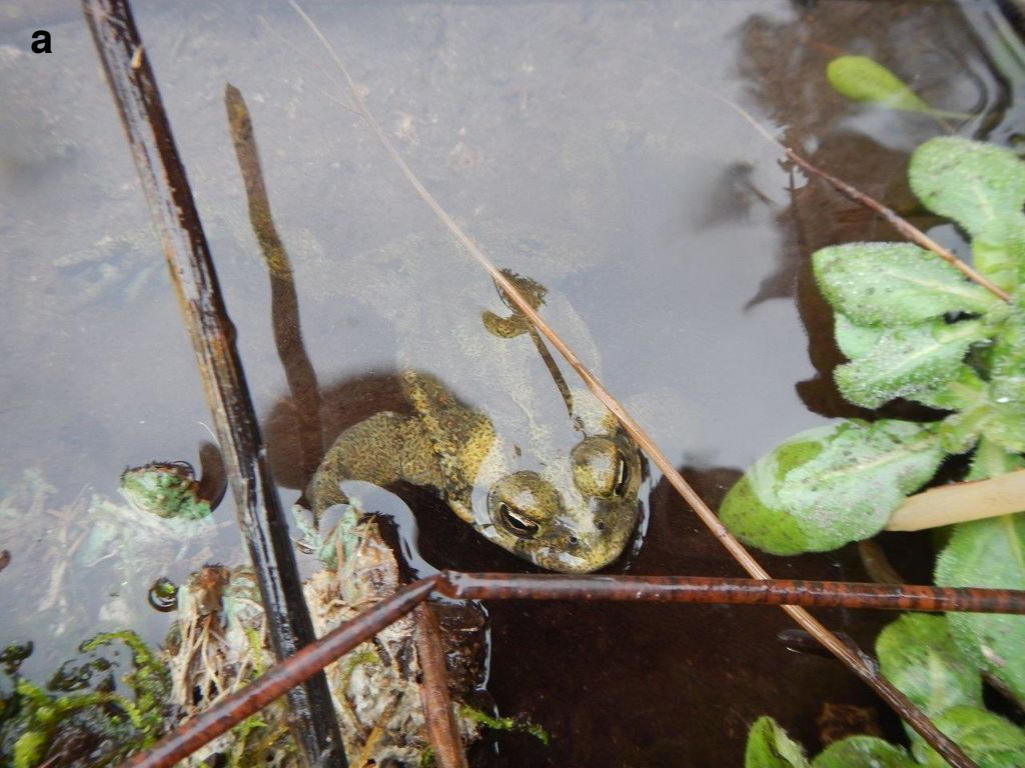


*Thermacarus* mites are apparently hot spring specialists as all four known species have been collected in waters of at least 40ºC (Sokolow 1927, Marshall 1928, Mitchell 1963, Nyquist 1965, Martin and Schwoerbel 2002). Like many water mites, adult *Thermacarus*, including *T. nevadensis*, are predators of chironomid fly larvae (Mitchell 1960) and presumably will eat eggs of Ephydridae (Collins et al. 1976). The larvae of *Thermacarus* appear to be unique among Parasitengona (excluding chiggers) in parasitizing vertebrate hosts (Walter and Proctor 2013). The only known vertebrate host for the Thermacaridae is the toad *Rhinella spinulosa* (Wiegmann, 1834), confirmed as the host of *T. andinus* in South America (Martin and Schwoerbel 2002). Toads have also been suggested as the likely larval host of *T. nevadensis* (Smith and Cook 1991, Walter and Proctor 2013), though Martin and Schwoerbel (2002) indicate that this has not been confirmed for this species as the material identifed as *T. nevadensis* from Chile by Schwoerbel (1987) was in fact *T. andinus*. However, a toad host for *T. nevadensis* may still be likely as adult Western Toads (*Anaxyrus boreas* Baird and Girard, 1852) are frequently observed in the Alpha Stream and Delta/Epsilon and Gamma springs and thermal swamps of the park (Fig. 9), which suggest some tolerance of this amphibian to high temperatures for at least brief periods of time by adults, and possibly for the tadpoles (see Brues 1932); in fact, several amphibian species seem to show some tolerance to higher water temperatures (Brues 1928, Brues 1932, Cunningham and Mullally 1956). For some amphibian species, exposure to hot water has been correlated with lower levels of chytrid fungal infection (Forrest and Schlaepfer 2011) which is known to be present in Western Toad populations in adjacent regions of Canada (Schock et al. 2010).

There is also some evidence that larvae of *T. nevadensis* may be attracted to other vertebrates, as larva have been found on, though not attached to humans in hot springs (Mitchell 1960). At least one species of fish is also known to inhabit some of these hot springs (McPhail 2001).

Viets (1938) also suggested that adult, winged insects that visit the hot spring pools may also serve as larval hosts; this also has not been confirmed for this species, but has for other hot spring mites (Wiegert and Mitchell 1973). As indicated by Martin and Schwoerbel (2002), larvae of *Thermacarus* mites could be "aerial" (after Mitchell 1957) to some degree (i.e., able to leave the water surface or go on shore) to reach potential hosts, whether invertebrate or vertebrate. Clearly, there is much to discover regarding the life history and hosts of *T. nevadensis*.

### Conservation in Canada

There are many hot spring habitats within British Columbia, particularly in the cordillera regions, but many of these are threatened by increased residiential and/or recreational development (Smith et al. 2011, Woodsworth and Woodsworth 2014). This is especially true in the more easily accessed areas of the southern Montane Cordillera (including the Western Interior Basin) (Smith et al. 2011). It is likely that these areas could, or could have, harboured at least two species of hot spring associated water mites, including *T. nevadensis* (and *Wandesia thermalis* (Viets, 1938)), both of which were considered common in similar habitats of the western United States (Smith et al. 2011), including Nevada (Marshall 1928, Mitchell 1963), Oregon (Mitchell 1963) and Colorado (Young 1969); Mitchell (1960) indicate that *T. nevadensis* was common in many hot springs in the western United States where daily temperatures ranged from 32°C to 48°C. Surprisingly, *T. nevadensis* has not been reported in the Montane Cordillera Ecozone of Canada (Smith et al. 2011), though the specimens reported here, from the Boreal Cordillera Ecozone of northern British Columbia support that this species could range throughout the entire cordilla regions of the province, and in hot spring habitats of adjacent Albera and the Yukon Territory. South of the Boreal Cordillera, the absence of these mites may be a result of degradation of hot spring habitats for recreational use during the past century (Smith et al. 2011), though the species seems to be common in the spa of Liard River hotsprings complex, located within Liard River Hotsprings Provincial Park, which is also home to an at risk endemic snail species (Heron 2007). It is also likely that many hot springs in the cordillera regions of Canada have not been extensively sampled. At present, and like some of the other at risk hot spring invertebrates in Canada (e.g., Heron 2007, COSEWIC 2008), *T. nevadensis* may be geographically restricted to more remote, undeveloped and/or protected sites of northeastern British Columbia. Interestingly for *T. nevadensis*, there are very slight (0.46% maximum distance), albeit consistent differences in DNA barcodes from mites from the two populations studied (i.e., Alpha Pool and Grayling River), suggesting that these two populations are likely isolated from each other, which warrants further comparisons between these sites and populations elsewhere in North America.

From a conservation perspective, the presence of *Thermacarus* mites in Canada has evolutionary and ecological significance. As indicated by Martin and Schwoerbel (2002), these are the only water mites whose larvae parasitize amphibians (though this is not yet confirmed for *T. nevadensis*), and other than chiggers (Trombiculidae and Leeuwenhoekiidae), *Thermacarus* are one of the few groups with larval parasitengone which feed on vertebrates (Walter and Proctor 2013). As such, *T. nevadensis* can be considered a unique member of the Canadian fauna with very specialized habitat requirements. Efforts to document its full distribution in Canada, including its specific thermal and chemical tolerances, should be undertaken.

## Supplementary Material

XML Treatment for Thermacarus nevadensis

## Figures and Tables

**Figure 6a.**
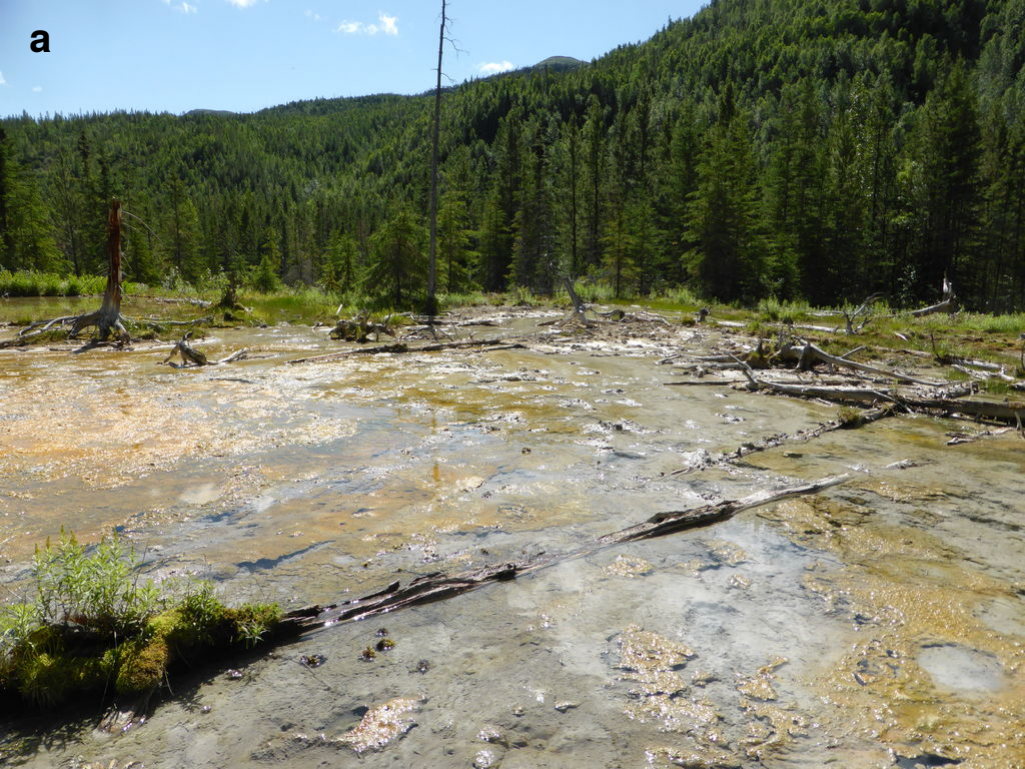
Hot spring at ground level.

**Figure 6b.**
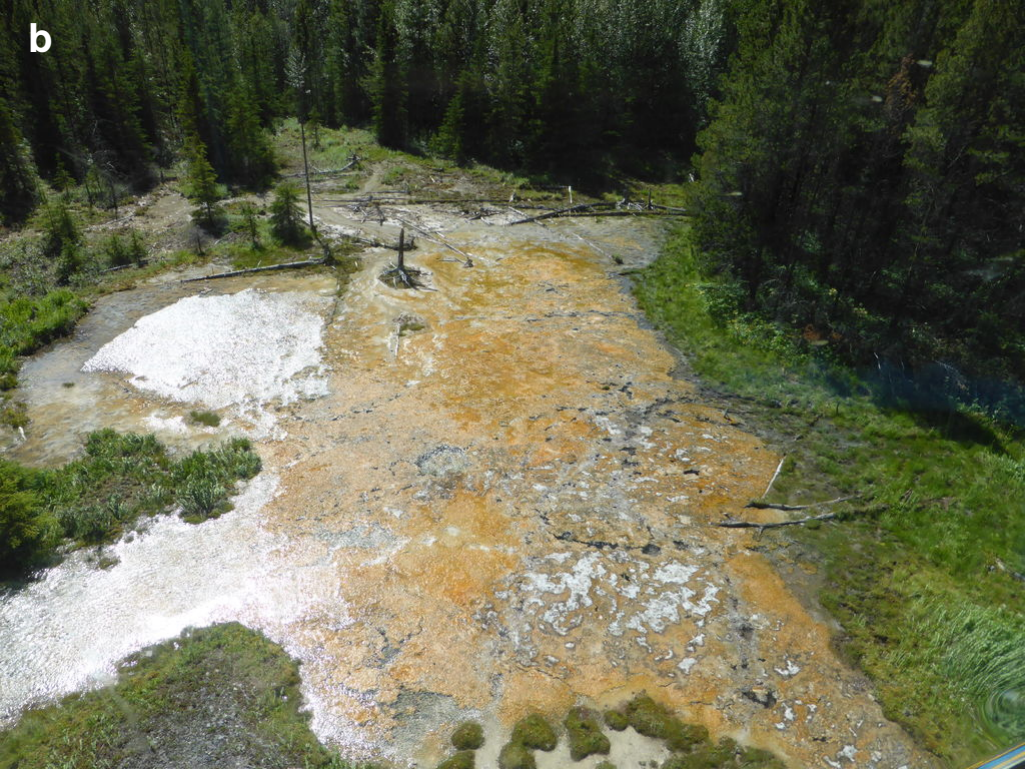
Hot spring viewed from above.

**Figure 8a.**
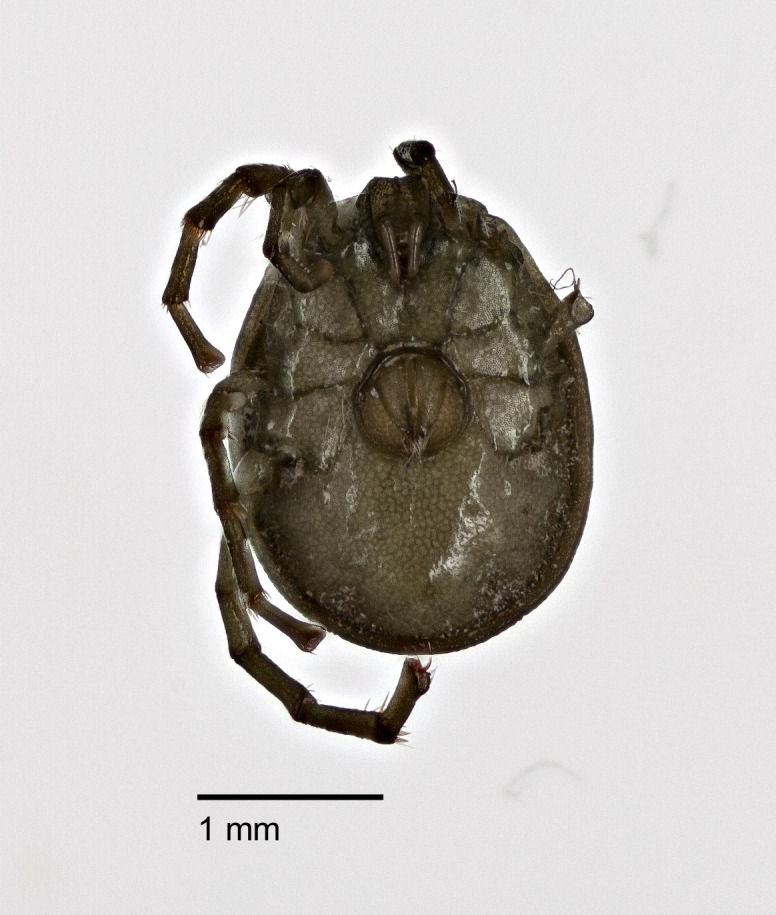
Female, ventral view; some of the legs removed to facilitate DNA barcoding and photography.

**Figure 8b.**
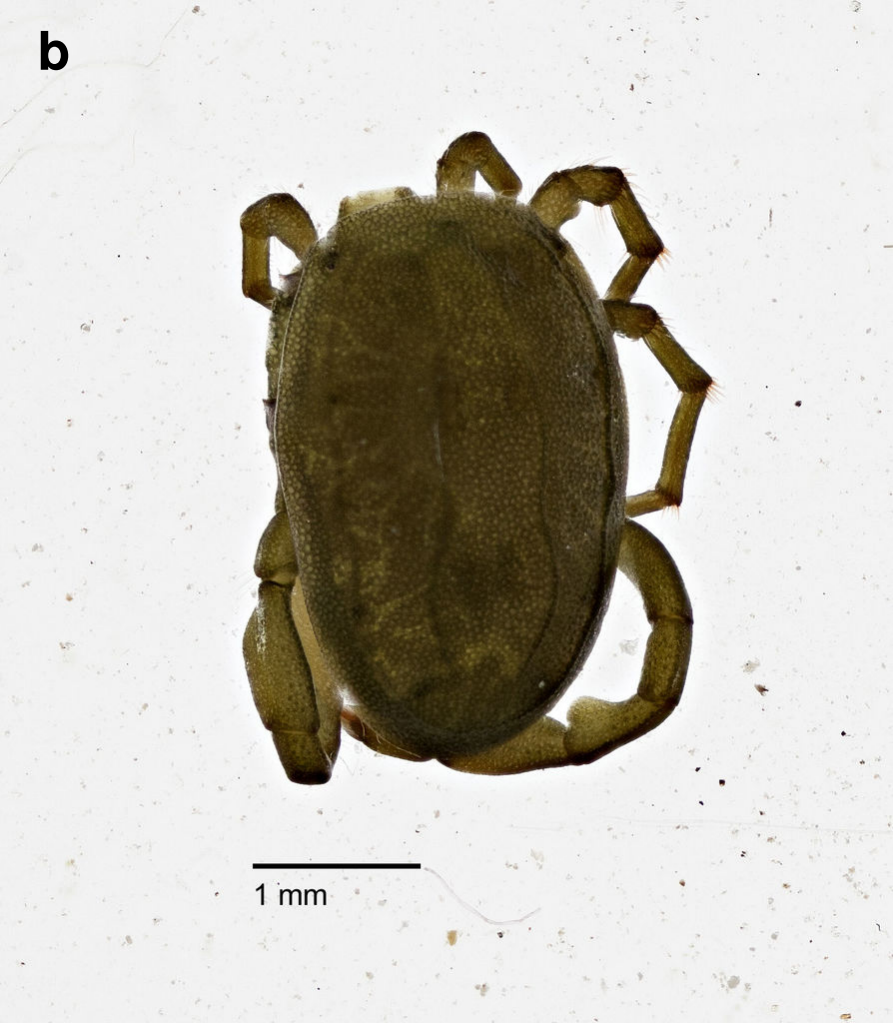
Male, dorsal view.

**Figure 9b.**